# Szász-Durrmeyer operators involving Boas-Buck polynomials of blending type

**DOI:** 10.1186/s13660-017-1396-x

**Published:** 2017-05-23

**Authors:** Manjari Sidharth, PN Agrawal, Serkan Araci

**Affiliations:** 1 Department of Mathematics, Indian Institute of Technology Roorkee, Roorkee, 247667 India; 2 Department of Economics, Faculty of Economics, Administrative and Social Sciences, Hasan Kalyoncu University, Gaziantep, 27410 Turkey

**Keywords:** 41A10, 41A25, 41A36, Lipschitz class function, Ditzian-Totik modulus of smoothness, weighted modulus of continuity

## Abstract

The present paper introduces the Szász-Durrmeyer type operators based on Boas-Buck type polynomials which include Brenke type polynomials, Sheffer polynomials and Appell polynomials considered by Sucu et al. (Abstr. Appl. Anal. 2012:680340, 2012). We establish the moments of the operator and a Voronvskaja type asymptotic theorem and then proceed to studying the convergence of the operators with the help of Lipschitz type space and weighted modulus of continuity. Next, we obtain a direct approximation theorem with the aid of unified Ditzian-Totik modulus of smoothness. Furthermore, we study the approximation of functions whose derivatives are locally of bounded variation.

## Introduction

For a real-valued bounded function *f* on $[0,1]$, Bernstein [2] defined a sequence of polynomials given by

$$\begin{aligned} B_{n}(f;x)=\sum_{r=0}^{n}f \biggl( \frac{r}{n} \biggr) {\binom{n}{r}}x^{r}(1-x)^{n-r}, \quad \forall x\in [0,1]\mbox{ and } n\in \mathbb{N}, \end{aligned}$$

to provide a very simple and elegant proof of the Weierstrass approximation theorem. For $f\in C[0,\infty )$, Szász [3] generalized the Bernstein polynomials to the infinite interval as follows:

$$\begin{aligned} S_{n}(f;x)=e^{-nx}\sum_{k=0}^{\infty } \frac{(nx)^{k}}{k!}f \biggl( \frac{k}{n} \biggr) , \quad \forall x\in [0, \infty )\mbox{ and } n\in \mathbb{N}, \end{aligned}$$

provided the infinite series on the right-hand side converges. Recently, the Szász operators, their quantum and post quantum analogues, Szász-Durrmeyer operators and mixed type operators have been intensively studied. We refer the readers to the related papers (cf. [4–9] etc.).

In [1], Sucu et al. introduced the Szász operators involving Boas-Buck type polynomials as follows:

1.1 $$\begin{aligned} B_{n}(f;x):=\frac{1}{A(1)G(n x H(1))}\sum _{k=0}^{\infty }p_{k}(nx)f \biggl( \frac{k}{n} \biggr) , \quad x\geq 0, n\in \mathbb{N}, \end{aligned}$$

where a generating function of the Boas-Buck type polynomials is given by

1.2 $$ A(t)G \bigl(x H(t) \bigr)=\sum_{k=0}^{\infty }p_{k}(x)t^{k}, $$

and $A(t)$, $G(t)$ and $H(t)$ are analytic functions described as

$$\begin{aligned}& A(t)=\sum_{k=0}^{\infty }a_{k} t^{k}\quad (a_{0}\neq 0),\qquad G(t)=\sum_{k=0}^{\infty }g_{k} t^{k}\quad (g_{k}\neq 0), \\& H(t)=\sum_{k=1}^{\infty }h_{k} t^{k}\quad (h_{1}\neq 0). \end{aligned}$$

Motivated by the above work, in the present paper we define Szász-Durrmeyer type operators based on Boas-Buck type polynomials as follows.

For $\gamma >0$, let $C_{\gamma }[0,\infty ):= \{ f\in C[0,\infty ): \vert f(t) \vert \leq M (1+t^{\gamma })\mbox{ for some } M>0 \} $ endowed with the norm

$$\Vert f \Vert _{\gamma }=\sup_{t\in [0,\infty )}\frac{\vert f(t) \vert }{(1+t^{\gamma })}. $$

Then, for a function $f\in C_{\gamma }[0,\infty )$, we define

1.3 $$\begin{aligned} M_{n}(f;x)&:=\frac{1}{A(1)G(n x H(1))}\sum _{k=1}^{\infty } \frac{p_{k}(nx)}{B(k,n+1)} \int_{0}^{\infty } \frac{t^{k-1}}{(1+t)^{n+k+1}}f(t)\,dt \\ &\quad {}+\frac{a_{0}b_{0}}{A(1)G(n x H(1))}f(0), \end{aligned}$$

where $B(k,n+1)$ is the beta function and $x\geq 0$, $n\in \mathbb{N}$.

Alternatively, we may write operator (1.3) as

1.4 $$\begin{aligned} M_{n}(f;x):= \int_{0}^{\infty }W(n,x,t)f(t)\,dt, \end{aligned}$$

where

$$\begin{aligned} W(n,x,t):={}&\frac{1}{A(1)G(n x H(1))}\sum_{k=1}^{\infty } \frac{p_{k}(nx)}{B(k,n+1)}\frac{t^{k-1}}{(1+t)^{n+k+1}} \\ &{}+\frac{a_{0}b _{0}}{A(1)G(n x H(1))}\delta (t), \end{aligned}$$

and $\delta (t)$ is the Dirac-delta function.

We study the approximation properties of the operators $M_{n}$ for functions belonging to different function spaces.

## Preliminaries

### Lemma 1

[1]

*For the operators* $B_{n}$, *one has*

$$\begin{aligned} \mathrm{(i)} \quad& B_{n}(1;x)=1 , \\ \mathrm{(ii)}\quad &B_{n}(s;x)=\frac{G^{\prime }(nxH(1))}{G(nxH(1))}x+\frac{A^{\prime }(1)}{nA(1)} , \\ \mathrm{(iii)}\quad &B_{n} \bigl(s^{2};x \bigr)= \frac{G^{\prime \prime }(nxH(1))}{G(nxH(1))}x^{2}+\frac{ ( 2 A^{ \prime }(1)+(1+H^{\prime \prime }(1))A(1) ) }{n A(1)}\frac{G^{\prime }(nxH(1))}{G(nxH(1))}x \\ &\hphantom{B_{n}(s^{2};x)={}}{} +\frac{A^{\prime \prime }(1)+A(1)}{n^{2} A(1)} , \\ \mathrm{(iv)}\quad &B_{n} \bigl(s^{3};x \bigr)= \frac{G^{\prime \prime \prime }(nxH(1))}{G(nxH(1))}x^{3}+\frac{ ( 3A(1)+3A^{\prime }(1)+3A(1)H^{\prime \prime }(1) ) }{n A(1)}\frac{G^{\prime \prime }(nxH(1))}{G(nxH(1))}x^{2} \\ &\hphantom{B_{n}(s^{2};x)={}}{}+\frac{ ( 6A^{\prime }(1)+A(1)+3A(1)H^{\prime \prime }(1)+3A^{\prime \prime }(1)+3A^{\prime }(1)H^{\prime \prime }(1)+A(1)H^{\prime\prime \prime }(1) ) }{n^{2} A(1)} \\ &\hphantom{B_{n}(s^{2};x)={}}{}\times\frac{G^{\prime }(nxH(1))}{G(nxH(1))}x+\frac{4 A^{\prime \prime }(1)+A^{\prime }(1)}{n^{3} A(1)} , \\ \mathrm{(v)} \quad &B_{n} \bigl(s^{4};x \bigr)=\frac{G^{\mathrm{iv}}(nxH(1))}{G(nxH(1))}x^{4}+ \biggl(\frac{4A^{\prime }(1)+6A(1)H^{\prime \prime }(1)+6A(1)}{n A(1)} \biggr) \frac{G^{\prime \prime \prime }(nxH(1))}{G(nxH(1))}x^{3} \\ &\hphantom{B_{n}(s^{2};x){}}{}+ \biggl(\frac{6A^{\prime \prime }(1)+12 A^{\prime }(1)H^{\prime \prime }(1)+4A(1)H^{\prime \prime \prime }(1)+3A(1)(H^{\prime \prime }(1))^{2}+7A(1)+18A^{\prime }(1)+18A(1)H^{\prime \prime }(1)}{n^{2}A(1)} \biggr) \\ &\hphantom{B_{n}(s^{2};x){}}{}\times\frac{G^{\prime \prime}(nxH(1))}{G(nxH(1))}x^{2} \\ &\hphantom{B_{n}(s^{2};x){}} {}+ \biggl(\frac{4A^{\prime \prime \prime }(1)+6A^{\prime \prime }(1)H^{\prime \prime }(1) +4A^{\prime }(1)H^{\prime \prime \prime }(1)+A(1)H^{\mathrm{iv}}(1)+36A^{\prime }(1)}{n^{3} A(1)} \\ &\hphantom{B_{n}(s^{2};x){}}{}+\frac{A(1)+7A(1)H^{\prime \prime }(1)+18A^{\prime \prime }(1)+18A^{\prime }(1)H^{\prime \prime }(1)+6A(1)H^{\prime \prime \prime }(1)-22A^{\prime }(1)}{n^{3} A(1)} \biggr) \\ &\hphantom{B_{n}(s^{2};x){}}{}{}\times\frac{G^{\prime }(nxH(1))}{G(nxH(1))}x+ \biggl( \frac{13 A^{\prime \prime }(1)+A^{\prime }(1)+A^{\mathrm{iv}}}{A(1)} \biggr) . \end{aligned}$$

### Proof

Since identities (i)-(iii) are proved in [1], we give below the proof of only (iv). Identity (v) follows similarly.

It is easily seen that

$$\begin{aligned} &\sum_{k=0}^{\infty }k^{3}p_{k}(nx) \\ &\quad= \bigl(4A^{\prime \prime }(1)+A^{\prime }(1) \bigr)G \bigl(nxH(1) \bigr) \\ &\qquad {}+ \bigl(6A^{\prime }(1)+A(1)+3A(1)H^{\prime \prime }(1)+3A^{\prime \prime }(1)+3A^{\prime }(1)H^{\prime \prime }(1) \\ &\qquad {}+A(1)H^{\prime \prime \prime }(1) \bigr)G^{\prime } \bigl(nxH(1) \bigr)nx+ \bigl(3A(1)+3A ^{\prime }(1)+3A(1)H^{\prime \prime }(1) \bigr)G^{\prime \prime } \bigl(nxH(1) \bigr)n ^{2} x^{2} \\ &\qquad {}+A(1)G^{\prime \prime \prime } \bigl(nxH(1) \bigr)n^{3} x^{3}, \end{aligned}$$

and

$$\begin{aligned} &\sum_{k=0}^{\infty }k^{4} p_{k}(nx) \\ &\quad =A(1)G^{\mathrm{iv}} \bigl(nxH(1) \bigr)n^{4} x^{4}+ \bigl(4A^{\prime }(1)+6A(1)H^{\prime \prime }(1)+6A(1) \bigr)G^{\prime \prime \prime } \bigl(nxH(1) \bigr)n^{3} x^{3} \\ &\quad \quad {}+ \bigl(6A^{\prime \prime }(1)+12 A^{\prime }(1)H^{\prime \prime }(1)+4A(1)H ^{\prime \prime \prime }(1)+3A(1) \bigl(H^{\prime \prime }(1) \bigr)^{2}+7A(1)+18A ^{\prime }(1) \\ &\quad \quad {}+18A(1)H^{\prime \prime }(1) \bigr)G^{\prime \prime } \bigl(nxH(1) \bigr)n^{2} x ^{2} + \bigl(4A^{\prime \prime \prime }(1)+6A^{\prime \prime }(1)H^{ \prime \prime }(1)+4A^{\prime }(1)H^{\prime \prime \prime }(1) \\ &\quad \quad {}+A(1)H^{\mathrm{iv}}(1)+36A^{\prime }(1)+A(1)+7A(1)H^{\prime \prime }(1) \\ &\quad \quad {}+18A ^{\prime \prime }(1)+18A^{\prime }(1)H^{\prime \prime }(1)+6A(1)H^{ \prime \prime \prime }(1) \\ &\quad \quad {}-22A^{\prime }(1) \bigr)G^{\prime } \bigl(nxH(1) \bigr)nx + \bigl(13A^{\prime \prime }(1)+A ^{\prime }(1)+A^{\mathrm{iv}}(1) \bigr)G \bigl(nxH(1) \bigr). \end{aligned}$$

 □

Now, by simple calculations, we obtain identities (iii) and (iv). Hence the details are omitted.

In the following lemma, we obtain the moments for the operators defined by (1.3) utilizing Lemma 1.

### Lemma 2

$$\begin{aligned} \mathrm{(i)} \quad & M_{n}(1;x)=1 , \\ \mathrm{(ii)} \quad & M_{n}(t;x)=\frac{1}{n} \biggl( \frac{G^{\prime }(nxH(1))}{G(nxH(1))}nx+\frac{A^{ \prime }(1)}{A(1)} \biggr) , \\ \mathrm{(iii)} \quad& M_{n} \bigl(t^{2};x \bigr)= \frac{1}{n(n-1)} \biggl[\frac{G^{\prime \prime }(nxH(1))}{G(nxH(1))}n ^{2}x^{2}+ \biggl( 2\frac{A^{\prime }(1)}{A(1)}+H^{\prime \prime }(1)+2 \biggr) \frac{G^{\prime }(nxH(1))}{G(nxH(1))}nx \\ &\hphantom{M_{n} (t^{2};x )=}{}+2\frac{A^{\prime }(1)}{A(1)}+\frac{A ^{\prime \prime }(1)}{A(1)} \biggr] , \\ \mathrm{(iv)} \quad &M_{n} \bigl(t^{3};x \bigr)= \frac{1}{n(n-1)(n-2)} \biggl[ \frac{G^{\prime \prime \prime }(nxH(1))}{G(nxH(1))}n^{3}x^{3} \\ &\hphantom{M_{n} (t^{2};x )=}{}+ \biggl(3\frac{A^{\prime }(1)}{A(1)}+6+3H^{\prime \prime }(1) \biggr) \frac{G^{\prime \prime }(nxH(1))}{G(nxH(1))}n^{2}x^{2} \\ &\hphantom{M_{n} (t^{2};x )=}{}+ \biggl(12\frac{A ^{\prime }(1)}{A(1)}+H^{\prime \prime }(1)+3 \frac{A^{\prime }(1)}{A(1)}H^{\prime \prime }(1)+H^{\prime \prime\prime }(1)+4 \biggr) \frac{G^{\prime }(nxH(1))}{G(nxH(1))}nx \\ &\hphantom{M_{n} (t^{2};x )=}{}+ 7\frac{A ^{\prime \prime }(1)}{A(1)}+6\frac{A^{\prime }(1)}{A(1)} \biggr] , \\ \mathrm{(v)} \quad &M_{n} \bigl(t^{4};x \bigr)= \frac{1}{n(n-1)(n-2)(n-3)} \biggl[\frac{G^{\mathrm{iv}}(nxH(1))}{G(nxH(1))}n^{4}x ^{4} \\ &\hphantom{M_{n} (t^{2};x )=}{}+ \biggl(4\frac{A^{\prime }(1)}{A(1)} +6H^{\prime \prime }(1)+12 \biggr) \frac{G^{\prime \prime \prime }(nxH(1))}{G(nxH(1))}n^{3}x^{3} \\ &\hphantom{M_{n} (t^{2};x )=}{}+ \biggl(6\frac{A^{\prime \prime }(1)}{A(1)}+12 \frac{A^{\prime }(1)}{A(1)}H^{\prime \prime }(1)+21 \frac{A^{\prime }(1)}{A(1)}+3\frac{H^{\prime \prime }(1)}{A(1)} \\ &\hphantom{M_{n} (t^{2};x )=}{}+4H ^{\prime \prime \prime }(1)+18H^{\prime \prime }(1)+3 \bigl(H^{\prime \prime }(1) \bigr)^{2}+21 \biggr)\frac{G^{\prime \prime }(nxH(1))}{G(nxH(1))}n^{2}x^{2} \\ &\hphantom{M_{n} (t^{2};x )=}{}+ \biggl(4\frac{A^{\prime \prime }(1)}{A(1)}+6 \frac{A^{\prime\prime }(1)}{A(1)}H^{\prime \prime }(1)+36 \frac{A^{\prime \prime }(1)}{A(1)}H^{\prime \prime }(1)+42 \frac{A^{ \prime }(1)}{A(1)} \\ &\hphantom{M_{n} (t^{2};x )=}{}+4 \frac{A^{\prime }(1)}{A(1)}H^{\prime \prime\prime }(1)+36 \frac{A^{\prime \prime }(1)}{A(1)}+H^{\mathrm{iv}}(1)+12H^{\prime \prime \prime }(1)+36H^{\prime \prime }(1)+24 \biggr) \\ &\hphantom{M_{n} (t^{2};x )=}{}\times\frac{G^{ \prime }(nxH(1))}{G(nxH(1))}nx+\frac{A^{\mathrm{iv}}(1)}{A(1)}+48 \frac{A^{\prime \prime }(1)}{A(1)}+13 \frac{A^{\prime }(1)}{A(1)}+11 \biggr] . \end{aligned}$$

Hence, as a consequence of Lemma 2, we find the following.

### Lemma 3

*For operator* (1.3), *we have the following results*:

$$\begin{aligned} \mathrm{(i)} \quad &M_{n} \bigl((t-x);x \bigr)= \biggl( \frac{G^{\prime }(nxH(1))}{G(nxH(1))}-1 \biggr) x+\frac{A^{\prime}(1)}{nA(1)} , \\ \mathrm{(ii)} \quad &M_{n} \bigl((t-x)^{2};x \bigr)=\biggl(\frac{n}{n-1}\frac{G^{\prime \prime }(nxH(1))}{G(nxH(1))}-2\frac{G ^{\prime }(nxH(1))}{G(nxH(1))}+1\biggr)x^{2} \\ &\hphantom{M_{n} ((t-x)^{2};x )=}{} + \biggl(\frac{1}{n-1} \biggl( 2\frac{A ^{\prime }(1)}{A(1)}+H^{\prime \prime }(1)+2\biggr) \frac{G^{\prime }(nxH(1))}{G(nxH(1))}-\frac{2}{n}\frac{A^{ \prime }(1)}{A(1)} \biggr)x \\ &\hphantom{M_{n} ((t-x)^{2};x )=}{} + \frac{1}{n(n-1)} \biggl( 2\frac{A^{\prime }(1)}{A(1)}+ \frac{A^{\prime \prime }(1)}{A(1)} \biggr) , \\ \mathrm{(iii)} \quad &M_{n} \bigl((t-x)^{4};x \bigr)= \biggl\{ \frac{n^{3}}{(n-1)(n-2)(n-3)}\frac{G^{\mathrm{iv}}(nxH(1))}{G(nxH(1))} \\ &\hphantom{M_{n} ((t-x)^{2};x )=}{} -\frac{4n^{2}}{(n-1)(n-2)}\frac{G^{\prime \prime \prime }(nxH(1))}{G(nxH(1))}+ \frac{6n}{(n-1)} \frac{G^{\prime \prime }(nxH(1))}{G(nxH(1))} \\ &\hphantom{M_{n} ((t-x)^{2};x )=}{} -4\frac{G^{\prime }(nxH(1))}{G(nxH(1))}+1 \biggr\} x^{4} \\ &\hphantom{M_{n} ((t-x)^{2};x )=}{} + \biggl\{ \frac{n^{2}}{(n-1)(n-2)(n-3)} \frac{G^{\prime \prime \prime }(nxH(1))}{G(nxH(1))} \biggl(4 \frac{A^{\prime }(1)}{A(1)}+6 H^{\prime \prime }(1)+12 \biggr) \\ &\hphantom{M_{n} ((t-x)^{2};x )=}{}- \frac{4n}{(n-1)(n-2)} \frac{G^{\prime \prime }(nxH(1))}{G(nxH(1))} \biggl(3 \frac{A^{\prime }(1)}{A(1)}+3 H^{\prime \prime }(1)+6 \biggr) \\ &\hphantom{M_{n} ((t-x)^{2};x )=}{}+\frac{6}{(n-1)}\frac{G^{\prime }(nxH(1))}{G(nxH(1))} \biggl(2 \frac{A^{\prime }(1)}{A(1)}+H^{\prime \prime }(1)+2 \biggr)- \frac{4}{n} \frac{A^{\prime }(1)}{A(1)} \biggr\} x^{3} \\ &\hphantom{M_{n} ((t-x)^{2};x )=}{}+ \biggl\{ \frac{n}{(n-1)(n-2)(n-3)}\frac{G^{\prime \prime }(nxH(1))}{G(nxH(1))} \biggl(6 \frac{A^{\prime \prime }(1)}{A(1)}+12 \frac{A^{\prime }(1)}{A(1)}H^{\prime \prime }(1) \\ &\hphantom{M_{n} ((t-x)^{2};x )=}{}+21\frac{A^{\prime }(1)}{A(1)}+3\frac{H^{\prime \prime }(1)}{A(1)}+4H ^{\prime \prime \prime }(1)+18H^{\prime \prime }(1)+3 \bigl(H^{\prime \prime }(1) \bigr)^{2}+21 \biggr) \\ &\hphantom{M_{n} ((t-x)^{2};x )=}{}- \frac{4}{(n-1)(n-2)}\frac{G^{\prime }(nxH(1))}{G(nxH(1))} \biggl(12 \frac{A^{\prime }(1)}{A(1)}+H^{\prime \prime }(1)+3 \frac{A^{\prime }(1)}{A(1)}H^{\prime \prime }(1) \\ &\hphantom{M_{n} ((t-x)^{2};x )=}{}+H^{\prime \prime }(1)+4 \biggr) \biggr\} x^{2}+ \biggl\{ \frac{1}{(n-1)(n-2)(n-3)}\frac{G^{\prime }(nxH(1))}{G(nxH(1))} \biggl(4\frac{A^{\prime \prime }(1)}{A(1)} \\ &\hphantom{M_{n} ((t-x)^{2};x )=}{}+6\frac{A^{\prime \prime }(1)}{A(1)}H^{\prime \prime }(1)+36 \frac{A^{\prime\prime }(1)}{A(1)}H^{\prime \prime }(1)+42 \frac{A^{\prime }(1)}{A(1)}+4 \frac{A^{\prime }(1)}{A(1)}H^{\prime \prime \prime }(1) \\ &\hphantom{M_{n} ((t-x)^{2};x )=}{}+36\frac{A^{\prime \prime }(1)}{A(1)}+H^{\mathrm{iv}}(1)+12H^{\prime \prime \prime }(1)+36H ^{\prime \prime }(1)+24 \biggr) \\ &\hphantom{M_{n} ((t-x)^{2};x )=}{}- \frac{4}{n(n-1)(n-2)} \biggl(7\frac{A^{\prime \prime }(1)}{A(1)}+6 \frac{A^{\prime }(1)}{A(1)} \biggr) \biggr\} x \\ &\hphantom{M_{n} ((t-x)^{2};x )=}{}+\frac{1}{(n-1)(n-2)(n-3)} \biggl(\frac{A^{\mathrm{iv}}(1)}{A(1)}+48 \frac{A ^{\prime \prime }(1)}{A(1)}+13\frac{A^{\prime }(1)}{A(1)}+11 \biggr) . \end{aligned}$$

Now, in order to study the approximation properties of the considered operators (1.3), we make the following assumptions on the analytic functions $A(t)$, $H(t)$ and $G(t)$. It is to be noted that the following assumptions are valid pointwise. These assumptions will be needed to prove Theorems 3, 7 and 8 of this paper which are pointwise results.

$$\begin{aligned} &\lim_{n\rightarrow \infty }n \biggl\{ \frac{G^{\prime }(nxH(1))}{G(nxH(1))}-1 \biggr\} =l_{1}(x) , \\ & \lim_{n\rightarrow \infty }n \biggl\{ \frac{n}{n-1}\frac{G^{\prime \prime }(nxH(1))}{G(nxH(1))}-2 \frac{G^{\prime }(nxH(1))}{G(nxH(1))}+1 \biggr\} =l_{2}(x) , \\ &\lim_{n\rightarrow \infty }n^{2} \biggl\{ \frac{n^{2}}{(n-1)(n-2)(n-3)} \frac{G^{\prime \prime \prime }(nxH(1))}{G(nxH(1))} \biggl(4\frac{A^{ \prime }(1)}{A(1)}+6 H^{\prime \prime }(1)+12 \biggr) \\ &\quad {}- \frac{4n}{(n-1)(n-2)}\frac{G^{\prime \prime }(nxH(1))}{G(nxH(1))} \biggl(3\frac{A^{\prime }(1)}{A(1)}+3 H^{\prime \prime }(1)+6 \biggr) \\ &\quad {}+ \frac{6}{(n-1)}\frac{G^{\prime }(nxH(1))}{G(nxH(1))} \biggl(2 \frac{A^{\prime }(1)}{A(1)}+ H^{\prime \prime }(1)+2 \biggr)-\frac{4}{n}\frac{A^{\prime }(1)}{A(1)} \biggr\} =l_{3}(x) , \\ &\lim_{n\rightarrow \infty }n^{2} \biggl\{ \frac{n^{3}}{(n-1)(n-2)(n-3)} \frac{G^{\mathrm{iv}}(nxH(1))}{G(nxH(1))}-\frac{4n^{2}}{(n-1)(n-2)}\frac{G^{\prime \prime \prime }(nxH(1))}{G(nxH(1))} \\ &\quad {}+\frac{6n}{(n-1)}\frac{G^{\prime\prime }(nxH(1))}{G(nxH(1))}-4\frac{G^{\prime }(nxH(1))}{G(nxH(1))}+1 \biggr\} =l_{4}(x) . \end{aligned}$$

As a result of the above assumptions, applying Lemma 3, we reach the following important result.

### Lemma 4

*For operator* (1.3), *we have*

$$\begin{aligned} \mathrm{(i)} \quad & \lim_{n\rightarrow \infty }n M_{n}\bigl((t-x);x \bigr)=l_{1}(x)x+\frac{A^{\prime }(1)}{A(1)} , \\ \mathrm{(iii)} \quad &\lim_{n\rightarrow \infty }n M_{n} \bigl((t-x)^{2};x\bigr)=l_{2}(x)x^{2}+x \bigl(H^{ \prime \prime }(1)+2\bigr)=\eta (x)\quad (\textit{say}), \\ \mathrm{(iii)} \quad & \lim_{n\rightarrow \infty }n^{2}M_{n} \bigl((t-x)^{4};x\bigr) \\ &\quad =l_{4}(x)x^{4}+l _{3}(x)x^{3} \\ &\quad \quad {}+ \biggl(6\frac{A^{\prime \prime }(1)}{A(1)}-27 \frac{A^{\prime }(1)}{A(1)}+\frac{H^{\prime \prime }(1)}{A(1)} +14H^{\prime \prime }(1)+3\bigl(H^{\prime \prime }(1)\bigr)^{2}+5 \biggr) \\ &\quad =\nu (x)\quad (\textit{say}). \end{aligned}$$

## Results and discussion

Throughout the paper, we assume $\delta_{n}(x)=M_{n}((t-x)^{2};x)$.

In the following theorem, we show that the operators defined by (1.3) are an approximation process for $f\in C_{\gamma }[0, \infty )$, using the Bohman-Korovkin theorem.

### Theorem 1

*Let* $f\in C_{\gamma }[0,\infty )$. *Then*

$$ \lim_{n\rightarrow \infty }M_{n}(f;x)=f(x) $$

*holds uniformly in* $x\in [0,a]$, $a>0$.

### Proof

From Lemma 2, it follows that

$$ \lim_{n\rightarrow \infty }M_{n} \bigl(t^{i};x \bigr)=x^{i},\quad i=0,1,2, $$

uniformly in $x\in [0,a]$. Hence, by the Bohman-Korovkin theorem, the required result is immediate. □

First, we consider the Lipschitz type space [3] considered by Otto Szász to establish the uniform convergence of the Szász operators for functions in this space. For $0<\xi \leq 1$, $x\in (0,\infty )$, $t\in [0,\infty )$, we define

$$\begin{aligned} \operatorname{Lip}_{M}^{*}(\xi ):={}& \biggl\{ f\in C[0,\infty ): \bigl\vert f(t)-f(x) \bigr\vert \leq M_{f} \frac{\vert t-x \vert ^{ \xi }}{(t+x)^{\frac{\xi }{2}}}; \\ &{}\mbox{ where } M_{f} \mbox{ is a constant which depends on }f\biggr\} . \end{aligned}$$

In the following theorem, we find the rate of convergence of the operators $M_{n}$ for functions in $\operatorname{Lip}_{M}^{*}\xi $. We observe that due to the presence of *x*, in the denominator on the right-hand side, we get only pointwise approximation. In the case of Szász operators [3], this *x* gets canceled leading to the uniform convergence.

### Theorem 2

*Let* $f\in \operatorname{Lip}_{M}^{*}(\xi )$ *and* $\xi \in (0,1]$. *Then*, *for all* $x\in (0,\infty )$, *we have*

$$ \bigl\vert M_{n}(f;x)-f(x) \bigr\vert \leq M \biggl( \frac{\delta_{n}(x)}{x} \biggr)^{\frac{\xi }{2}}. $$

### Proof

By the linearity and positivity of the operators $M_{n}$, from (1.4) we obtain

$$ \bigl\vert M_{n}(f;x)-f(x) \bigr\vert \leq \int_{0}^{\infty } W(n,x,t) \bigl\vert f(t)-f(x) \bigr\vert \,dt. $$

Applying Hölder’s inequality with $p=\frac{2}{\xi } $ and $q=\frac{2}{2-\xi }$ and Lemma 2, we have

$$\begin{aligned} \bigl\vert M_{n}(f;x)-f(x) \bigr\vert \leq & \biggl( \int_{0}^{\infty }W(n,x,t) \bigl\vert f(t)-f(x) \bigr\vert ^{\frac{2}{ \xi }}\,dt \biggr)^{\frac{\xi }{2}} \biggl( \int_{0}^{\infty } W(n,x,t)\,dt \biggr)^{\frac{2-\xi }{2}} \\ \leq & \biggl( \int_{0}^{\infty }W(n,x,t) \bigl\vert f(t)-f(x) \bigr\vert ^{\frac{2}{\xi }}\,dt \biggr)^{\frac{\xi }{2}} \\ \leq & M \biggl( \int_{0}^{\infty }W(n,x,t)\frac{(t-x)^{2}}{(t+x)}\,dt \biggr)^{\frac{\xi }{2}} \\ \leq & M \biggl(\frac{\delta_{n}(x)}{x} \biggr)^{\frac{\xi }{2}}. \end{aligned}$$

Thus, we reach the desired result. □

In our next result, we establish a Voronovskaja type approximation theorem.

### Theorem 3

*Let* $f\in C_{\gamma }[0,\infty )$, *admitting a derivative of second order at a point* $x\in [0,\infty )$, *then there holds*

3.1 $$\begin{aligned} \lim_{n\rightarrow \infty }n \bigl(M_{n}(f;x)-f(x) \bigr) =& \biggl\{ l_{1}(x)x+\frac{A ^{\prime }(1)}{A(1)} \biggr\} f^{\prime }(x)+ \bigl\{ l_{2}(x)x^{2}+x \bigl(H ^{\prime \prime }(1)+2 \bigr) \bigr\} \frac{f^{\prime \prime }(x)}{2}. \end{aligned}$$

*If* $f^{\prime \prime }$ *is continuous on* $[0,\infty )$, *then the limit in* (3.1) *holds uniformly in* $x\in [0,a]\subset [0,\infty )$, $a>0$.

### Proof

By Taylor’s theorem

3.2 $$ f(t)=f(x)+f^{\prime }(x) (t-x)+\frac{1}{2}f^{\prime \prime }(x) (t-x)^{2}+ \varepsilon (t,x) (t-x)^{2}, $$

where $\varepsilon (t,x)\in C_{\gamma }[0,\infty )$ and $\lim_{t\rightarrow x}\varepsilon (t,x)=0$.

Applying the operator $M_{n}(\cdot,x)$ on both sides of (3.2), we have

3.3 $$\begin{aligned} \lim_{n\rightarrow \infty }n \bigl( M_{n}(f;x)-f(x) \bigr) =& \lim_{n\rightarrow \infty }n M_{n}(t-x;x)f^{\prime }(x) \\ &{} + \lim_{n\rightarrow \infty }n M_{n} \bigl((t-x)^{2};x \bigr)\frac{f^{\prime \prime }(x)}{2} \\ &{}+\lim_{n\rightarrow \infty }n M_{n} \bigl(\varepsilon (t,x) (t-x)^{2};x \bigr). \end{aligned}$$

Using the Cauchy-Schwarz inequality in the last term of the right-hand side of (3.3), we get

$$\begin{aligned} n M_{n} \bigl(\varepsilon (t,x) (t-x)^{2};x \bigr)\leq \sqrt{ M_{n} \bigl(\varepsilon ^{2}(t,x);x \bigr)} \sqrt{n^{2} M_{n} \bigl((t-x)^{4};x \bigr)}. \end{aligned}$$

Since $\varepsilon (t,x)\rightarrow 0$, as $t\rightarrow x$, applying Theorem 1, for every $x\in [0,\infty )$, we obtain $\lim_{n\rightarrow \infty }M_{n}(\varepsilon^{2}(t,x);x)=\varepsilon ^{2}(x,x)=0$. Next applying Lemma 4, for sufficiently large *n* and every $x\in [0,\infty )$, we have

3.4 $$ n^{2} M_{n} \bigl((t-x)^{4};x \bigr)=O(1). $$

Hence,

3.5 $$\begin{aligned} \lim_{n\rightarrow \infty } nM_{n} \bigl(\varepsilon (t,x) (t-x)^{2};x \bigr)=0. \end{aligned}$$

Now, from (3.3), (3.5) and Lemma 4, the required result follows. □

The uniformity assertion follows from the uniform continuity of $f^{\prime \prime }$ on $[0,a]$ and the fact that all the other estimates hold uniformly in $x\in [0,a]$.

In our next theorem, we obtain the degree of approximation of the $M_{n}$ operators for functions in the space $C_{2}[0,\infty )$ in terms of the classical modulus of continuity.

### Theorem 4

*For* $f\in C_{2}[0,\infty )$, *we have the following inequality*:

3.6 $$\begin{aligned} \bigl\vert M_{n}(f;x)-f(x) \bigr\vert \leq 4 M_{f} \bigl(1+x^{2} \bigr)\delta_{n}(x)+2 \omega_{b+1} \bigl( f;\sqrt{\delta_{n}(x)} \bigr) , \end{aligned}$$

*where* $\omega (f;\delta_{n}(x))$ *is the modulus of continuity of* *f* *on* $[0,b+1]$.

### Proof

From [10], for $t\in (b+1,\infty )$ and $x\in [0,b]$, we have

$$\begin{aligned} \bigl\vert f(t)-f(x) \bigr\vert \leq & 4 M_{f}(t-x)^{2} \bigl(1+x^{2} \bigr)+ \biggl( 1+\frac{\vert t-x \vert }{ \delta } \biggr) \omega_{b+1}(f,\delta ),\quad \delta >0. \end{aligned}$$

Hence, by applying the Cauchy-Schwarz inequality, we obtain

$$\begin{aligned} \bigl\vert M_{n}(f;x)-f(x) \bigr\vert \leq &4 M_{f} \bigl(1+x^{2} \bigr)M_{n} \bigl((t-x)^{2};x \bigr) \\ &{}+ \omega_{b+1}(f, \delta ) \biggl( 1+\frac{1}{\delta } \bigl(M_{n} \bigl((t-x)^{2};x \bigr) \bigr)^{1/2} \biggr) \\ =& M_{f} \bigl(1+x^{2} \bigr)\delta_{n}(x)+ \omega_{b+1}(f,\delta ) \biggl( 1+\frac{1}{ \delta }\sqrt{ \delta_{n}(x)} \biggr). \end{aligned}$$

Choosing $\delta =\sqrt{\delta_{n}(x)}$, we get the desired result. □

The next section is devoted to the weighted approximation properties of the operators $M_{n}$.

### Weighted approximation

Let

$$ C_{2}^{0}[0,\infty ):= \biggl\{ f\in C_{2}[0,\infty ); \lim_{x\rightarrow \infty }\frac{\vert f(x) \vert }{1+x^{2}} \mbox{ exists and is finite} \biggr\} . $$

Next, we study the approximation of functions in the subspace $C_{2}^{0}[0,\infty )$ of $C_{2}[0,\infty )$. Such type of function spaces has been considered by several researchers (cf. [11, 12]).

It is well known that the classical modulus of continuity of first order $\omega (f;\delta ),\delta >0 $ does not tend to zero, as $\delta \rightarrow 0$, on an infinite interval. A weighted modulus of continuity $\Omega (f;\delta ) $ which tends to zero as $\delta \rightarrow 0$ on $[0,\infty ) $ was defined in [13]. For $f\in C^{0}_{2}[0,\infty )$, the weighted modulus of continuity defined by Yüksel and Ispir [13] is given as follows:

3.7 $$ \Omega (f;\delta )=\sup_{x\in [0,\infty ), 0< h\leq \delta } \frac{\vert f(x+h)-f(x) \vert }{1+(x+h)^{2}}. $$

Some properties of $\Omega (f;\delta )$ are collected in the following lemma.

#### Lemma 5

[13]

*Let* $f\in C^{0}_{2}[0,\infty )$. *Then the following results hold*:

1. $\Omega (f;\delta )$ *is a monotonically increasing function of* *δ*;
2. $\lim_{\delta \rightarrow 0^{+}}\Omega (f;\delta )=0$;
3. *For each* $m\in \mathbb{N}$, $\Omega (f;m\delta )\leq m\Omega (f;\delta)$;
4. *For each* $\lambda \in (0,\infty ), \Omega (f;\lambda \delta )\leq (1+\lambda ) \Omega (f;\delta )$.

Firstly, we establish the following basic approximation theorem for functions in the weighted space of continuous functions $C^{0}_{2}[0, \infty )$ by the operators $M_{n}$.

#### Theorem 5

*For* $f\in C^{0}_{2}[0,\infty )$ *and* $a>0$, *we have*

$$\begin{aligned} \lim_{n\rightarrow \infty }\sup_{x\in [0,\infty )}\frac{\vert M_{n}(f;x)-f(x)\vert }{(1+x ^{2})^{1+a}}=0. \end{aligned}$$

#### Proof

Let $x_{0}\in [0,\infty )$ be an arbitrary but fixed point. Then

3.8 $$\begin{aligned} &\sup_{x\in [0,\infty )} \frac{\vert M_{n}(f;x)-f(x)\vert }{(1+x^{2})^{1+a}} \\ &\quad \leq \sup_{x\leq x_{0}} \frac{\vert M_{n}(f;x)-f(x)\vert }{(1+x^{2})^{1+a}}+\sup _{x>x_{0}}\frac{ \vert M_{n}(f;x)-f(x)\vert }{(1+x^{2})^{1+a}} \\ &\quad \leq \bigl\Vert M_{n}(f;\cdot)-f \bigr\Vert _{C[0,x_{0}]}+ \Vert f \Vert _{2}\sup_{x>x_{0}}\frac{M_{n}(1+t^{2};x)}{(1+x^{2})^{1+a}}+\sup_{x>x_{0}}\frac{\vert f(x) \vert }{(1+x^{2})^{1+a}}. \end{aligned}$$

Since $\vert f(x) \vert \leq \Vert f \Vert _{2}(1+x^{2})$, we have

$$ \sup_{x>x_{0}}\frac{\vert f(x) \vert }{(1+x^{2})^{1+a}}\leq \frac{\Vert f \Vert _{2}}{(1+x _{0}^{2})^{a}}. $$

Let $\epsilon >0$ be arbitrary. We choose $x_{0}$ to be so large that

3.9 $$ \frac{\Vert f \Vert _{2}}{(1+x_{0}^{2})^{a}}< \frac{\epsilon }{6}\quad \mbox{so that } \sup _{x>x_{0}}\frac{\vert f(x) \vert }{(1+x^{2})^{1+a}}< \frac{ \epsilon }{6}. $$

From Theorem 1, there exists $n_{1}\in \mathbb{N}$ such that

$$\begin{aligned} \Vert f \Vert _{2}\frac{M_{n}(1+t^{2};x)}{(1+x^{2})^{1+a}} \leq &\frac{\Vert f \Vert _{2}}{(1+x ^{2})^{a}} \biggl( 1+x^{2}+\frac{\epsilon }{3\Vert f \Vert _{2}} \biggr),\quad \forall n>n_{1} \\ \leq & \frac{\Vert f \Vert _{2}}{(1+x_{0}^{2})^{a}}+\frac{\epsilon }{3},\quad \forall n>n_{1} \mbox{ and } x>x_{0}. \end{aligned}$$

Hence,

3.10 $$\begin{aligned} \Vert f \Vert _{2}\sup_{x>x_{0}} \frac{M_{n}(1+t^{2};x)}{(1+x^{2})^{1+a}} \leq \frac{\epsilon }{2}, \quad \forall n>n_{1}. \end{aligned}$$

Applying Theorem 3, we can find $n_{2}\in \mathbb{N}$ such that

3.11 $$\begin{aligned} \bigl\Vert M_{n}(f;\cdot)-f \bigr\Vert _{C[0,x_{0}]}< \frac{\epsilon }{3}\quad \forall n>n _{2}. \end{aligned}$$

Let $n_{0}=\max (n_{1},n_{2})$. Combining (3.8)-(3.11), we obtain

$$ \sup_{x\in [0,\infty )} \frac{\vert M_{n}(f;x)-f(x)\vert }{(1+x^{2})^{1+a}}< \epsilon ,\quad \forall n>n_{0}. $$

Hence the required result is obtained. □

In our next theorem, we determine the order of approximation for functions in a weighted space of continuous functions on $[0,\infty )$ by $M_{n}$ operators.

#### Theorem 6

*Let* $f\in C^{0}_{2}[0,\infty )$. *Then*, *for sufficiently large* *n*, *we have*

3.12 $$ \bigl\vert M_{n}(f;x)-f(x) \bigr\vert \leq C(x) \Omega \biggl( f;\frac{1}{ \sqrt{n}} \biggr) , $$

*where* $C(x)=2(1+x^{2}) (1+C_{1}\vert \eta (x) \vert +\sqrt{C_{1}}\vert \eta (x) \vert ^{1/2} ( 1+\sqrt{C_{2}}\vert \nu (x) \vert ^{1/2} ) ), C_{1}, C_{2}$ *are constants independent of* *x* *and* *n* *and* $\eta (x)$, $\nu (x)$ *are as given in Lemma* 4.

#### Proof

For $x\in (0,\infty )$ and $\delta >0$, using (3.7) and Lemma 5, we have

$$\begin{aligned} \bigl\vert f(t)-f(x) \bigr\vert &\leq \bigl( 1+ \bigl(x+\vert x-t \vert \bigr)^{2} \bigr) \Omega \bigl(f;\vert t-x \vert \bigr) \\ &\leq 2 \bigl(1+x^{2} \bigr) \bigl(1+(t-x)^{2} \bigr) \biggl( 1+\frac{\vert t-x \vert }{\delta } \biggr) \Omega (f;\delta ). \end{aligned}$$

Applying $M_{n}(\cdot ;x)$ on both sides, we can write

3.13 $$\begin{aligned} & \bigl\vert M_{n}(f;x)-f(x) \bigr\vert \\ &\quad \leq 2 \bigl(1+x^{2} \bigr)\Omega (f;\delta ) \biggl(1+M_{n} \bigl((t-x)^{2};x \bigr)+M _{n} \biggl( \bigl(1+(t-x)^{2} \bigr)\frac{\vert t-x \vert }{\delta };x \biggr) \biggr). \end{aligned}$$

From Lemma 4, for sufficiently large *n*, it follows

3.14 $$\begin{aligned} n M_{n} \bigl((t-x)^{2};x \bigr)\leq C_{1}\bigl\vert \eta (x) \bigr\vert \quad \mbox{and}\quad n^{2}M_{n} \bigl((t-x)^{4};x \bigr)\leq C_{2}\bigl\vert \nu (x) \bigr\vert . \end{aligned}$$

Now, applying the Cauchy-Schwarz inequality in the last term of (3.13), we obtain

3.15 $$\begin{aligned} &M_{n} \biggl( \bigl(1+(t-x)^{2} \bigr) \frac{\vert t-x \vert }{\delta };x \biggr) \\ &\quad \leq \frac{1}{ \delta } \bigl( M_{n} \bigl((t-x)^{2};x \bigr) \bigr) ^{1/2}+\frac{1}{\delta } \bigl( M _{n} \bigl((t-x)^{4};x \bigr) \bigr) ^{1/2} \bigl( M_{n} \bigl((t-x)^{2};x \bigr) \bigr) ^{1/2}. \end{aligned}$$

Combining the estimates (3.13)-(3.15) and taking

$$ \delta =\frac{1}{\sqrt{n}}, $$

we reach the required result. □

### Unified modulus theorem

We investigate a direct approximation theorem by utilizing the unified Ditzian-Totik modulus of smoothness $\omega_{\phi^{\tau }}(f,t), 0 \leq \tau \leq 1$. Guo et al. [14] proved the direct, inverse and equivalence approximation theorems with the aid of unified modulus. First, we give the definitions of the Ditzian-Totik modulus of smoothness and the Peetre’s *K*-functional. Let $\phi^{2}(x)=x(1+x)$ and $f\in C_{B}[0,\infty )$, the space of all bounded and continuous functions on $[0,\infty )$ endowed with the norm $\Vert f \Vert = \sup_{x\in [0,\infty )}\vert f(x) \vert $. The modulus $\omega_{\phi^{\tau }}(f,t)$, $0\leq \tau \leq 1$, is defined as

$$\begin{aligned} \omega_{\phi^{\tau }}(f,t)= \sup_{0\leq h \leq t} \sup _{x\pm \frac{h\phi^{\tau }(x)}{2}\in [0,\infty )} \biggl\vert f \biggl( x+\frac{h\phi^{\tau }(x)}{2} \biggr) -f \biggl( x-\frac{h\phi^{\tau }(x)}{2} \biggr) \biggr\vert , \end{aligned}$$

and the appropriate *K*-functional is given by

$$ K_{\phi^{\tau }}(f,t)=\inf_{g\in W_{\tau }} \bigl\{ \Vert f-g \Vert +t \bigl\Vert \phi^{\tau }g^{\prime } \bigr\Vert \bigr\} , $$

where $W_{\tau }=\{g:g\in A C_{\mathrm{loc}}[0,\infty ):\Vert \phi^{\tau }g^{\prime } \Vert <\infty \}$, $A C_{\mathrm{loc}}$ denotes the space of locally absolutely continuous functions on $[0,\infty )$.

From [15], there exists a constant $M>0$ such that

3.16 $$\begin{aligned} M^{-1}\omega_{\phi^{\tau }}(f,t)\leq K_{\phi^{\tau }}(f,t)\leq M \omega_{\phi^{\tau }}(f,t). \end{aligned}$$

#### Theorem 7

*Let* $f\in C_{B}[0,\infty )$, *then for sufficiently large* *n*

$$\begin{aligned} \bigl\vert M_{n}(f;x)-f(x) \bigr\vert \leq & C \omega_{\phi^{\tau }} \biggl( f;\frac{ \phi^{1-\tau }(x)}{\sqrt{n}} \biggr) , \end{aligned}$$

*where* *C* *is independent of* *f* *and* *n*.

#### Proof

By the definition of $K_{\phi^{\tau }}(f,t)$, for fixed *n*, *x*, *τ*, we can choose $g=g_{n,x,\tau }\in W_{\tau }$ such that

3.17 $$\begin{aligned} \Vert f-g \Vert +\frac{\phi^{1-\tau }(x)}{\sqrt{n}} \bigl\Vert \phi^{\tau }g^{\prime } \bigr\Vert \leq 2 K_{\phi^{\tau }} \biggl( f;\frac{\phi^{1-\tau }(x)}{\sqrt{n}} \biggr). \end{aligned}$$

We may write

3.18 $$\begin{aligned} \bigl\vert M_{n}(f;x)-f(x) \bigr\vert &\leq \bigl\vert M_{n}(f-g;x) \bigr\vert + \bigl\vert M_{n}(g;x)-g(x) \bigr\vert + \bigl\vert g(x)-f(x) \bigr\vert \\ &\leq 2 \Vert f-g \Vert + \bigl\vert M_{n}(g;x)-g(x) \bigr\vert . \end{aligned}$$

Since $g\in W_{\tau }$, we have

$$ g(t)=g(x)+ \int_{x}^{t}g^{\prime }(u)\,du $$

and so

3.19 $$\begin{aligned} \bigl\vert M_{n}(g;x)-g(x) \bigr\vert \leq & M_{n} \biggl( \biggl\vert \int_{x}^{t}g^{\prime }(u)\,du \biggr\vert ;x \biggr). \end{aligned}$$

By applying Hölder’s inequality, we get

$$\begin{aligned} \biggl\vert \int_{x}^{t}g^{\prime }(u)\,du \biggr\vert \leq & \bigl\Vert \phi^{\tau }g^{\prime } \bigr\Vert \biggl\vert \int_{x}^{t}\frac{du}{\phi^{\tau }(u)} \biggr\vert \leq \bigl\Vert \phi^{\tau }g^{\prime } \bigr\Vert \vert t-x \vert ^{1-\tau } \biggl\vert \int_{x} ^{t}\frac{du}{\phi (u)} \biggr\vert ^{\tau }, \end{aligned}$$

we may write

$$\begin{aligned} \biggl\vert \int_{x}^{t}\frac{du}{\phi (u)} \biggr\vert \leq & \biggl\vert \int_{x}^{t}\frac{du}{\sqrt{u}} \biggr\vert \biggl(\frac{1}{\sqrt{1+x}}+\frac{1}{ \sqrt{1+t}} \biggr). \end{aligned}$$

Hence, on using the inequality $\vert a+b \vert ^{r}\leq \vert a \vert ^{r}+\vert b \vert ^{r}$, $0\leq r\leq 1$.

3.20 $$\begin{aligned} \biggl\vert \int_{x}^{t}g^{\prime }(u)\,du \biggr\vert \leq & \frac{2^{\tau }\Vert \phi^{\tau }g^{\prime } \Vert \vert t-x \vert }{x^{\tau /2}} \biggl(\frac{1}{ \sqrt{1+x}}+\frac{1}{\sqrt{1+t}} \biggr)^{\tau } \\ \leq & \frac{2^{\tau }\Vert \phi^{\tau }g^{\prime } \Vert \vert t-x \vert }{x^{\tau /2}} \biggl(\frac{1}{(1+x)^{ \tau /2}}+\frac{1}{(1+t)^{\tau /2}} \biggr). \end{aligned}$$

Thus, from (3.19), (3.20) and the Cauchy-Schwarz inequality, using Theorem 1, we obtain

3.21 $$\begin{aligned} \bigl\vert M_{n}(g;x)-g(x) \bigr\vert \leq & \frac{2^{\tau }\Vert \phi^{\tau }g^{\prime } \Vert }{x ^{\tau /2}} M_{n} \biggl( \vert t-x \vert \biggl( \frac{1}{(1+x)^{\tau /2}}+\frac{1}{(1+t)^{ \tau /2}} \biggr);x \biggr) \\ \leq & \frac{2^{\tau }\Vert \phi^{\tau }g^{\prime } \Vert }{x^{\tau /2}} \biggl(\frac{1}{(1+x)^{\tau /2}}\sqrt{M_{n}\bigl((t-x)^{2};x \bigr)} \\ &{}+ \sqrt{M _{n} \bigl((t-x)^{2};x \bigr)}\sqrt{M_{n} \bigl( (1+t)^{-\tau };x \bigr) } \biggr) \\ \leq & 2^{\tau } \bigl\Vert \phi^{\tau }g^{\prime } \bigr\Vert \sqrt{M_{n} \bigl((t-x)^{2};x \bigr)} \bigl\{ \phi^{-\tau }(x)+x^{-\tau /2} \sqrt{M _{n} \bigl((1+t)^{-\tau };x \bigr) } \bigr\} \\ \leq & 2^{\tau }C \bigl\Vert \phi^{\tau }g^{\prime } \bigr\Vert \frac{\phi (x)}{ \sqrt{n}} \bigl\{ \phi^{-\tau }(x)+x^{-\tau /2}(1+x)^{-\tau /2} \bigr\} \\ =& 2^{\tau +1}\frac{\Vert \phi^{\tau }g^{\prime } \Vert \phi^{1-\tau }(x)}{ \sqrt{n}} \end{aligned}$$

for sufficiently large *n*.

Hence, combining (3.17)-(3.19) and (3.21), we find

$$\begin{aligned} \bigl\vert M_{n}(f;x)-f(x) \bigr\vert \leq & 2\Vert f-g \Vert +2^{\tau +1}C \bigl\Vert \phi^{\tau }g^{\prime} \bigr\Vert \frac{\phi^{1-\tau }}{\sqrt{n}} \\ \leq & C \biggl\{ \Vert f-g \Vert +\frac{\phi^{1-\tau }(x)}{\sqrt{n}} \bigl\Vert \phi^{\tau }g^{\prime } \bigr\Vert \biggr\} \\ \leq & 2 C K_{\phi^{\tau }} \biggl( f;\frac{\phi^{1-\tau }(x)}{ \sqrt{n}} \biggr) \\ \leq & C \omega_{\phi^{\tau }} \biggl( f;\frac{\phi^{1-\tau }(x)}{ \sqrt{n}} \biggr). \end{aligned}$$

This completes the proof of the theorem. □

### Rate of convergence of Szász-Durrmeyer operators based on Boas-Buck polynomials

In this section, we discuss the approximation of functions with a derivative of bounded variation. We show that the points *x* where $f^{\prime }(x+)$ and $f^{\prime }(x-)$ exist, the operators $M_{n}(f;x)$ converge to the function $f(x)$, as $n\rightarrow \infty $. In the recent years, several researchers have studied different sequences of linear positive operators. We refer the reader to some of the related papers (cf. [8, 9, 16–20] and [21] etc.). Let $DBV[0,\infty )$ be the class of all functions in $C_{2}[0,\infty )$ having a derivative which is locally of bounded variation on $[0,\infty )$. A function $f\in DBV[0,\infty )$ can be represented as

$$ f(x)= \int_{0}^{x} g(t)+f(0), $$

where *g* is a function of bounded variation on each finite subinterval of $[0,\infty )$.

#### Lemma 6

*Let* $\alpha =\alpha (n)\rightarrow 0$, *as* $n\rightarrow \infty $ *and* $\lim_{n\rightarrow \infty }n \alpha (n)=l\in \mathbb{R}$. *For all* $x\in (0,\infty )$ *and sufficiently large* *n*, *we have*

$$\begin{aligned} \mathrm{(i)}\quad & \xi_{n}(x,t)=\int_{0}^{t} W(n,x,t)\,du\leq \frac{C_{1} \vert \eta (x) \vert }{(x-t)^{2}} , \\ \mathrm{(ii)} \quad & 1-\xi_{n}(x,t)=\int_{t}^{\infty }W(n,x,t)\,du \leq \frac{C_{1}\vert \eta (x) \vert }{(t-x)^{2}} , \end{aligned}$$

*where* $\eta (x)$ *is as given in Lemma* 4.

#### Proof

Using Lemma 2 and (3.14), we have

$$\begin{aligned} \xi_{n}(x,t) &= \int_{0}^{t} W(n,x,t)\,du \\ &\leq \int_{0}^{t} \biggl( \frac{x-u}{x-t} \biggr)^{2} W(n,x,t) (x,u)\,du \\ &\leq \frac{1}{(x-t)^{2}}M_{n} \bigl((u-x)^{2};x \bigr) \\ &\leq \frac{C_{1}\vert \eta (x) \vert }{(x-t)^{2}}, \end{aligned}$$

when *n* is large enough. Similarly, we can prove (ii). □

#### Theorem 8

*Let* $f\in DB V[0,\infty )$. *Then*, *for every* $x\in (0,\infty )$ *and sufficiently large* *n*, *we have*

$$\begin{aligned} & \bigl\vert M_{n}(f;x)-f(x) \bigr\vert \\ &\quad \leq \biggl[ \biggl( \frac{G^{\prime }(nxH(1))}{G(nxH(1))}-1 \biggr) x+\frac{A ^{\prime }(1)}{nA(1)} \biggr] \biggl\vert \frac{f^{\prime }(x+)+f^{\prime }(x-)}{2} \biggr\vert \\ &\quad \quad {}+\sqrt{C_{1} \bigl\vert \eta (x) \bigr\vert } \biggl\vert \frac{f^{\prime }(x+)-f^{\prime }(x-)}{2} \biggr\vert + \frac{C_{1}\vert \eta (x) \vert }{x}\sum _{k=1}^{[\sqrt{n}]} \Biggl( \bigvee _{x-\frac{x}{k}}^{x} f^{\prime }_{x} \Biggr) \\ &\quad \quad {}+\frac{x}{\sqrt{n}} \Biggl(\bigvee_{x-\frac{x}{\sqrt{n}}}^{x} f ^{\prime }_{x} \Biggr)+ \biggl(4M_{f}+ \frac{M_{f}+\vert f(x) \vert }{x^{2}} \biggr)C _{1} \bigl\vert \eta (x) \bigr\vert + \bigl\vert f^{\prime }(x+) \bigr\vert \sqrt{C_{1} \bigl\vert \eta (x) \bigr\vert } \\ &\quad \quad {}+{\frac{C_{1}\vert \eta (x) \vert }{x^{2}}} \bigl\vert f(2x)\!-\!f(x)\!-\!x f^{\prime }(x+) \bigr\vert +\frac{x}{\sqrt{n}} \Biggl( \bigvee ^{x+\frac{x}{\sqrt{n}}}_{x} f^{\prime }_{x} \Biggr) + \frac{C _{1}\vert \eta (x) \vert }{x}\sum_{k=1}^{[\sqrt{n}]} \Biggl( \bigvee^{x+\frac{x}{\sqrt{n}}}_{x} f^{\prime }_{x} \Biggr), \end{aligned}$$

*where* $C_{1}$ *is a positive constant and* $\bigvee_{a}^{b} f$ *denotes the total variation of* *f* *on* $[a,b]$ *and* $f^{\prime }_{x}$ *is defined by*

3.22 $$ f^{\prime }_{x}(t)= \textstyle\begin{cases} f^{\prime }(t)-f^{\prime }(x-), & 0\leq t< x, \\ 0, & t=x, \\ f^{\prime }(t)-f^{\prime }(x+), & x< t< \infty. \end{cases} $$

#### Proof

For any $f\in DBV [0,\infty )$, from (3.22), we may write

3.23 $$\begin{aligned} f^{\prime }(u) =&\frac{1}{2} \bigl( f^{\prime }(x+)+f^{\prime }(x-) \bigr) +f ^{\prime }_{x}(u)+ \frac{1}{2} \bigl( f^{\prime }(x+)-f^{\prime }(x-) \bigr) \operatorname{sgn}(u-x) \\ &{}+\delta_{x}(u) \biggl( f^{\prime }(u)-\frac{1}{2} \bigl(f^{\prime }(x+)+f ^{\prime }(x-) \bigr) \biggr) , \end{aligned}$$

where

$$\begin{aligned} \delta_{x}(u)= \textstyle\begin{cases} 1, & u=x, \\ 0, & u\neq x. \end{cases}\displaystyle \end{aligned}$$

Since $M_{n}(e_{0};x)=1$, using (3.23) for every $x\in (0,\infty )$, we get

3.24 $$\begin{aligned} M_{n}(f;x)-f(x) =& \int_{0}^{\infty } W(n,x,t) \bigl(f(t)-f(x) \bigr)\,dt \\ =& \int_{0}^{\infty } W(n,x,t) \biggl( \int_{x}^{t} f^{\prime }(u)\,du \biggr)\,dt \\ =& - \int_{0}^{x} \biggl( \int_{t}^{x} f^{\prime }(u)\,du \biggr) W(n,x,t)\,dt \\ &{}+ \int_{x}^{\infty } \biggl( \int_{x}^{t} f^{\prime }(u)\,du \biggr) W(n,x,t)\,dt. \end{aligned}$$

Let

$$\begin{aligned}& I_{1}:= \int_{0}^{x} \biggl( \int_{t}^{x}f^{\prime }(u)\,du \biggr) W(n,x,t)\,dt, \\& I_{2}:= \int_{x}^{\infty } \biggl( \int_{x}^{t} f^{\prime }(u)\,du \biggr) W(n,x,t)\,dt. \end{aligned}$$

Since $\int_{x}^{t} \delta_{x}(u)\,du=0$, using (3.23), we have

3.25 $$\begin{aligned} I_{1} =& \int_{0}^{x} \biggl\{ \int_{t}^{x} \biggl(\frac{1}{2} \bigl( f^{ \prime }(x+)+f^{\prime }(x-) \bigr) +f^{\prime }_{x}(u) \\ &{}+\frac{1}{2} \bigl( f^{\prime }(x+)-f^{\prime }(x-) \bigr) \operatorname{sgn}(u-x) \biggr)\,du \biggr\} W(n,x,t)\,dt \\ =&\frac{1}{2} \bigl( f^{\prime }(x+)+f^{\prime }(x-) \bigr) \int_{0} ^{x} (x-t)W(n,x,t)\,dt+ \int_{0}^{x} \biggl( \int_{t}^{x} f^{\prime }_{x}(u)\,du \biggr) W(n,x,t)\,dt \\ &{}-\frac{1}{2} \bigl( f^{\prime }(x+)-f^{\prime }(x-) \bigr) \int_{0} ^{x} (x-t)W(n,x,t)\,dt. \end{aligned}$$

Similarly, we have

3.26 $$\begin{aligned} I_{2} =& \int_{x}^{\infty } \biggl\{ \int^{t}_{x} \biggl(\frac{1}{2} \bigl( f ^{\prime }(x+)+f^{\prime }(x-) \bigr) +f^{\prime }_{x}(u) \\ &{}+\frac{1}{2} \bigl( f^{\prime }(x+)-f^{\prime }(x-) \bigr) \operatorname{sgn}(u-x) \biggr)\,du \biggr\} W(n,x,t)\,dt \\ =&\frac{1}{2} \bigl( f^{\prime }(x+)+f^{\prime }(x-) \bigr) \int_{x} ^{\infty } (t-x)W(n,x,t)\,dt+ \int_{x}^{\infty } \biggl( \int_{x}^{t} f ^{\prime }_{x}(u)\,du \biggr) W(n,x,t)\,dt \\ &{}+\frac{1}{2} \bigl( f^{\prime }(x+)-f^{\prime }(x-) \bigr) \int_{x} ^{\infty } (t-x)W(n,x,t)\,dt. \end{aligned}$$

Combining relations (3.24)-(3.26), we get

$$\begin{aligned} M_{n}(f;x)-f(x)={}&\frac{1}{2} \bigl( f^{\prime }(x+)+f^{\prime }(x-) \bigr) \int_{0}^{\infty } (t-x)W(n,x,t)\,dt \\ &{}+\frac{1}{2} \bigl( f^{\prime }(x+)-f^{\prime }(x-) \bigr) \int_{0}^{\infty } \vert t-x \vert W(n,x,t)\,dt \\ &{}- \int_{0}^{x} \biggl( \int_{t}^{x}f^{\prime }_{x}(u)\,du \biggr) W(n,x,t)\,dt+ \int_{x}^{\infty } \biggl( \int_{x}^{t} f^{\prime }_{x}(u)\,du \biggr) W(n,x,t)\,dt. \end{aligned}$$

Hence,

3.27 $$\begin{aligned} & \bigl\vert M_{n}(f;x)-f(x) \bigr\vert \\ &\quad \leq \biggl\vert \frac{f^{\prime }(x+)+f^{\prime }(x-)}{2} \biggr\vert \bigl\vert M_{n}(t-x;x) \bigr\vert + \biggl\vert \frac{f^{\prime }(x+)-f^{\prime }(x-)}{2} \biggr\vert M_{n} \bigl( \vert t-x \vert ;x \bigr) \\ &\quad \quad {}+ \biggl\vert \int_{0}^{x} \biggl( \int_{t}^{x} f^{\prime }_{x}(u)\,du \biggr) W(n,x,t)\,dt \biggr\vert + \biggl\vert \int_{x}^{\infty } \biggl(\int_{x}^{t} f^{\prime }_{x}(u)\,du\biggr) W(n,x,t)\,dt \biggr\vert . \end{aligned}$$

Now, assume that

$$ C_{n} \bigl(f^{\prime }_{x},x \bigr)= \int_{0}^{x} \biggl( \int_{t}^{x}f^{\prime }_{x}(u)\,du \biggr) W(n,x,t)\,dt $$

and

$$ D_{n} \bigl(f^{\prime }_{x},x \bigr)= \int_{x}^{\infty } \biggl( \int_{x}^{t} f^{\prime }_{x}(u)\,du \biggr) W(n,x,t)\,dt. $$

Now the problem is reduced to estimate $C_{n}(f^{\prime }_{x},x)$ and $D_{n}(f^{\prime }_{x},x)$. Using the definition of $\xi_{n}(x,t)$ given in Lemma 6 and applying integration by parts, we can write

$$\begin{aligned} C_{n} \bigl(f^{\prime }_{x},x \bigr) &= \int_{0}^{x} \biggl( \int_{t}^{x} f^{\prime } _{x}(u)\,du \biggr) \frac{\partial \xi_{n}(x,t)}{\partial t}\,dt= \int_{0} ^{x}f^{\prime }_{x}(t) \xi_{n}(x,t)\,dt. \end{aligned}$$

Thus,

$$\begin{aligned} \bigl\vert C_{n} \bigl(f^{\prime }_{x},x \bigr) \bigr\vert &= \int_{0}^{x} \bigl\vert f^{\prime }_{x}(t) \bigr\vert \xi_{n}(x,t)\,dt \leq \int_{0}^{x-\frac{x}{\sqrt{n}}} \bigl\vert f^{\prime }_{x}(t) \bigr\vert \xi_{n}(x,t)\,dt+ \int_{x-\frac{x}{\sqrt{n}}}^{x} \bigl\vert f^{\prime }_{x}(t) \bigr\vert \xi_{n}(x,t)\,dt. \end{aligned}$$

Since $f^{\prime }_{x}(x)=0$ and $\xi_{n}(x,t)\leq 1$, we get

$$\begin{aligned} \int_{x-\frac{x}{\sqrt{n}}}^{x} \bigl\vert f^{\prime }_{x}(t) \bigr\vert \xi_{n}(x,t)\,dt &= \int_{x-\frac{x}{\sqrt{n}}}^{x} \bigl\vert f^{\prime }_{x}(t)-f^{\prime }_{x}(x) \bigr\vert \xi_{n}(x,t)\,dt\leq \int_{x-\frac{x}{\sqrt{n}}}^{x} \Biggl( \bigvee _{t}^{x} f^{\prime }_{x} \Biggr)\,dt \\ &\leq \Biggl( \bigvee_{x-\frac{x}{\sqrt{n}}}^{x} f^{\prime }_{x} \Biggr) \int_{x-\frac{x}{\sqrt{n}}}^{x}\,dt=\frac{x}{\sqrt{n}} \Biggl( \bigvee _{x-\frac{x}{ \sqrt{n}}}^{x} f^{\prime }_{x} \Biggr). \end{aligned}$$

Using Lemma 6 and assuming $t=x-\frac{x}{u}$, we have

$$\begin{aligned} \int^{x-\frac{x}{\sqrt{n}}}_{0} \bigl\vert f^{\prime }_{x}(t) \bigr\vert \xi_{n}(x,t)\,dt & \leq C_{1} \bigl\vert \eta (x) \bigr\vert \int^{x-\frac{x}{\sqrt{n}}}_{0}\frac{\vert f^{\prime }_{x}(t) \vert }{(x-t)^{2}}\,dt \\ &\leq C_{1}\bigl\vert \eta (x) \bigr\vert \int^{x-\frac{x}{\sqrt{n}}}_{0} \Biggl( \bigvee _{t}^{x} f^{\prime }_{x} \Biggr) \frac{dt}{(x-t)^{2}} \\ &= \frac{C_{1}\vert \eta (x) \vert }{ x} \int^{\sqrt{n}}_{1} \Biggl( \bigvee_{x-\frac{x}{u}}^{x} f^{\prime }_{x} \Biggr) \\ &\leq \frac{C_{1}\vert \eta (x) \vert }{ x}\sum_{k=1}^{[\sqrt{n}]} \Biggl( \bigvee_{x-\frac{x}{k}}^{x}f^{\prime }_{x} \Biggr). \end{aligned}$$

Therefore,

$$ \bigl\vert C_{n} \bigl(f^{\prime }_{x},x \bigr) \bigr\vert =\frac{C_{1}\vert \eta (x) \vert }{ x}\sum_{k=1}^{[ \sqrt{n}]} \Biggl( \bigvee_{x-\frac{x}{k}}^{x} f^{\prime }_{x} \Biggr) +\frac{x}{ \sqrt{n}} \Biggl( \bigvee _{x-\frac{x}{\sqrt{n}}}^{x} f^{\prime }_{x} \Biggr). $$

Using integration by parts in $D_{n}(f^{\prime }_{x},x)$ and applying Lemma 6, we have

$$\begin{aligned} \bigl\vert D_{n} \bigl(f^{\prime }_{x},x \bigr) \bigr\vert \leq & \biggl\vert \int_{x}^{2x} \biggl(\int_{x}^{t}f^{\prime }_{x}(u)\,du\biggr) \frac{\partial }{\partial t} \bigl(1-\xi_{n}(x,t) \bigr)\,dt \biggr\vert \\ &{}+ \biggl\vert \int_{2x}^{\infty } \biggl(\int_{x}^{t}f^{\prime }_{x}(u)\,du\biggr) W(n,x,t)\,dt \biggr\vert \\ \leq & \biggl\vert \int_{x}^{2x}f^{\prime }_{x}(u)\,du\bigg\vert \bigl\vert 1-\xi_{n}(x,2x) \bigr\vert + \int_{x}^{2x} \bigl\vert f^{\prime }_{x}(t)\bigr\vert \bigl(1-\xi_{n}(x,t) \bigr)\,dt \\ &{}+ \biggl\vert \int_{2x}^{\infty } \bigl(f(t)-f(x) \bigr)W(n,x,t)\,dt \biggr\vert \\ &{}+ \bigl\vert f^{\prime }(x+) \bigr\vert \biggl\vert \int_{2x}^{\infty }(t-x)W(n,x,t) (x,t)\,dt \biggr\vert . \end{aligned}$$

We have

3.28 $$\begin{aligned} \int_{x}^{2x} \bigl\vert f^{\prime }_{x}(t) \bigr\vert \bigl(1-\xi_{n}(x,t) \bigr)\,dt =& \int_{x}^{x+\frac{x}{ \sqrt{n}}} \bigl\vert f^{\prime }_{x}(t) \bigr\vert \bigl(1-\xi_{n}(x,t) \bigr)\,dt \\ &{}+ \int_{x+\frac{x}{\sqrt{n}}}^{2x} \bigl\vert f^{\prime }_{x}(t) \bigr\vert \bigl(1-\xi_{n}(x,t) \bigr)\,dt \\ =& J_{1}+J_{2}. \end{aligned}$$

Since $f^{\prime }_{x}(x)=0$ and $1-\xi_{n}(x,t)\leq 1$, we have

$$\begin{aligned} J_{1}= \int_{x}^{x+\frac{x}{\sqrt{n}}} \bigl\vert f^{\prime }_{x}(t)-f^{\prime}_{x}(x) \bigr\vert \bigl(1-\xi_{n}(x,t) \bigr)\,dt \leq \int_{x}^{x+\frac{x}{\sqrt{n}}} \Biggl( \bigvee _{x}^{x+\frac{x}{\sqrt{n}}}f^{\prime }_{x} \Biggr)\,dt= \frac{x}{ \sqrt{n}} \Biggl( \bigvee_{x}^{x+\frac{x}{\sqrt{n}}}f^{\prime }_{x} \Biggr). \end{aligned}$$

Using Lemma 6 and assuming $t=x+\frac{x}{u}$, we obtain

$$\begin{aligned} J_{2} &\leq C_{1}\bigl\vert \eta (x) \bigr\vert \int_{x+\frac{x}{\sqrt{n}}}^{2x} \frac{1}{(t-x)^{2}} \bigl\vert f^{\prime }_{x}(t)-f^{\prime }_{x}(x) \bigr\vert \,dt \leq C _{1} \bigl\vert \eta (x) \bigr\vert \int_{x+\frac{x}{\sqrt{n}}}^{2x}\frac{1}{(t-x)^{2}} \Biggl( \bigvee _{x}^{t} f^{\prime }_{x} \Biggr)\,dt \\ &= \frac{C_{1}\vert \eta (x) \vert }{ x} \int_{1}^{\sqrt{n}} \Biggl( \bigvee _{x} ^{x+\frac{x}{u}}f^{\prime }_{x} \Biggr)\,du \leq \frac{C_{1}\vert \eta (x) \vert }{ x}\sum_{k=1}^{[\sqrt{n}]} \int_{k}^{k+1} \Biggl( \bigvee _{x}^{x+ \frac{x}{u}}f^{\prime }_{x} \Biggr)\,du \\ &\leq \frac{C_{1}\vert \eta (x) \vert }{ x}\sum_{k=1}^{[\sqrt{n}]} \Biggl( \bigvee_{x} ^{x+\frac{x}{k}}f^{\prime }_{x} \Biggr). \end{aligned}$$

Putting the values of $J_{1}$ and $J_{2}$ in (3.28), we have

$$\begin{aligned} \int_{x}^{2x} \bigl\vert f^{\prime }_{x}(t) \bigr\vert \bigl(1-\xi_{n}(x,t) \bigr)\,dt &\leq \frac{x}{ \sqrt{n}} \Biggl( \bigvee_{x}^{x+\frac{x}{\sqrt{n}}}f^{\prime }_{x} \Biggr) +\frac{C _{1}\vert \eta (x) \vert }{ x}\sum_{k=1}^{[\sqrt{n}]} \Biggl( \bigvee_{x}^{x+ \frac{x}{k}}f^{\prime }_{x} \Biggr). \end{aligned}$$

Therefore, applying the Cauchy-Schwarz inequality and Lemma 6, we get

3.29 $$\begin{aligned} \bigl\vert D_{n} \bigl(f^{\prime }_{x},x \bigr) \bigr\vert \leq & M_{f} \int_{2x}^{\infty } \bigl(t^{2}+1 \bigr)W(n,x,t)\,dt+ \bigl\vert f(x) \bigr\vert \int_{2x}^{\infty }W(n,x,t)\,dt \\ &{}+ \bigl\vert f^{\prime }(x+) \bigr\vert \sqrt{C_{1}\bigl\vert \eta (x) \bigr\vert }+\frac{C_{1}\vert \eta (x) \vert }{ x^{2}} \bigl\vert f(2x)-f(x)-x f^{\prime }(x+) \bigr\vert \\ &{}+\frac{x}{\sqrt{n}} \Biggl( \bigvee_{x}^{x+\frac{x}{\sqrt{n}}}f ^{\prime }_{x} \Biggr) +\frac{C_{1}\vert \eta (x) \vert }{x}\sum _{k=1}^{[ \sqrt{n}]} \Biggl( \bigvee _{x}^{x+\frac{x}{k}}f^{\prime }_{x} \Biggr). \end{aligned}$$

Since $t\leq 2(t-x)$ and $x\leq t-x$ when $t\geq 2x$, we have

3.30 $$\begin{aligned} & M_{f} \int_{2x}^{\infty } \bigl(t^{2}+1 \bigr)W(n,x,t)\,dt+ \bigl\vert f(x) \bigr\vert \int_{2x}^{ \infty }W(n,x,t)\,dt \\ & \quad \leq \bigl(M_{f}+ \bigl\vert f(x) \bigr\vert \bigr) \int_{2x}^{\infty } W(n,x,t)\,dt +4M_{f} \int_{2x} ^{\infty }(t-x)^{2}W(n,x,t)\,dt \\ &\quad \leq \frac{M_{f}+\vert f(x) \vert }{x^{2}} \int_{0}^{\infty }(t-x)^{2}W(n,x,t)\,dt +4M_{f} \int_{0}^{\infty }(t-x)^{2}W(n,x,t)\,dt \\ &\quad \leq \biggl(4M_{f}+\frac{M_{f}+\vert f(x) \vert }{x^{2}} \biggr)C_{1}\bigl\vert \eta (x) \bigr\vert . \end{aligned}$$

Using the above inequality, we have

3.31 $$\begin{aligned} \bigl\vert D_{n} \bigl(f^{\prime }_{x},x \bigr) \bigr\vert \leq & \biggl(4M_{f}+\frac{M_{f}+\vert f(x) \vert }{x ^{2}} \biggr)C_{1}\bigl\vert \eta (x) \bigr\vert + \bigl\vert f^{\prime }(x+) \bigr\vert \sqrt{C_{1}\bigl\vert \eta (x) \bigr\vert } \\ &{}+ C_{1}\frac{1+x^{2}}{n x^{2}} \bigl\vert f(2x)-f(x)-x f^{\prime }(x+) \bigr\vert \\ &{}+\frac{x}{ \sqrt{n}} \Biggl( \bigvee_{x}^{x+\frac{x}{\sqrt{n}}}f^{\prime }_{x} \Biggr) +\frac{C_{1}\vert \eta (x) \vert }{ x}\sum_{k=1}^{[\sqrt{n}]} \Biggl( \bigvee_{x}^{x+\frac{x}{k}}f^{\prime }_{x} \Biggr). \end{aligned}$$

Now from (3.27), (3.29) and (3.31), we reach the required result. □

## Conclusion

We introduce Szász-Durremeyer type operators involving Boas-Buck type polynomials. Brenke type polynomials, Sheffer polynomials and Appell polynomials turn out to be the special cases of Boas-Buck type polynomials. We obtain the rate of convergence for functions belonging to a Lipschitz type space and also establish a Voronovskaja type theorem for twice continuously differentiable functions. We study the approximation properties of the considered operators for continuous functions in weighted spaces. Lastly, we discuss the rate of approximation of functions having derivatives of bounded variations.
